# Tropical butterflies use thermal buffering and thermal tolerance as alternative strategies to cope with temperature increase

**DOI:** 10.1111/1365-2656.13970

**Published:** 2023-07-12

**Authors:** Esme Ashe‐Jepson, Stephany Arizala Cobo, Yves Basset, Andrew J. Bladon, Irena Kleckova, Benita C. Laird‐Hopkins, Alex Mcfarlane, Katerina Sam, Amanda F. Savage, Ana Cecilia Zamora, Edgar C. Turner, Greg P. A. Lamarre

**Affiliations:** ^1^ Department of Zoology University of Cambridge Cambridge UK; ^2^ ForestGEO Smithsonian Tropical Research Institute Panama Republic of Panama; ^3^ Biology Centre of the Czech Academy of Sciences Institute of Entomology České Budějovice Czech Republic; ^4^ Faculty of Science University of South Bohemia České Budějovice Czech Republic; ^5^ Maestria de Entomologia University of Panama Panama Republic of Panama; ^6^ Smithsonian Tropical Research Institute Panama Republic of Panama

**Keywords:** buffering ability, butterfly, critical thermal maximum, ectotherm, insect, Lepidoptera, thermal ecology, thermal limits

## Abstract

1. Climate change poses a severe threat to many taxa, with increased mean temperatures and frequency of extreme weather events predicted.
2. Insects can respond to high temperatures using behaviour, such as angling their wings away from the sun or seeking cool local microclimates to thermoregulate or through physiological tolerance.
3. In a butterfly community in Panama, we compared the ability of adult butterflies from 54 species to control their body temperature across a range of air temperatures (thermal buffering ability), as well as assessing the critical thermal maxima for a subset of 24 species.
4. Thermal buffering ability and tolerance were influenced by family, wing length, and wing colour, with Pieridae, and butterflies that are large or darker in colour having the strongest thermal buffering ability, but Hesperiidae, small, and darker butterflies tolerating the highest temperatures.
5. We identified an interaction between thermal buffering ability and physiological tolerance, where species with stronger thermal buffering abilities had lower thermal tolerance, and vice versa. This interaction implies that species with more stable body temperatures in the field may be more vulnerable to increases in ambient temperatures, for example heat waves associated with ongoing climate change.
6. Our study demonstrates that tropical species employ diverse thermoregulatory strategies, which is also reflected in their sensitivity to temperature extremes.

## Graphical abstract

Tropical butterflies with a stronger ability to thermoregulate have lower thermal tolerance, and vice versa. This implies that species adapt to climate change using one strategy at the expense of the other, and many tropical butterflies may be at risk.

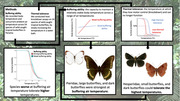

## Abstracta

1. El cambio climático representa una grave amenaza para muchos taxones, con un aumento de las temperaturas medias y la frecuencia de eventos climáticos extremos pronosticados.
2. Los insectos pueden responder a las altas temperaturas mediante comportamientos, como inclinar sus alas fuera del alcance del sol o buscar microclimas frescos locales para termorregular, o a través de la tolerancia fisiológica.
3. En una comunidad de mariposas en Panamá, comparamos la capacidad de las mariposas adultas de 54 especies para controlar su temperatura corporal en un rango de temperaturas del aire (capacidad de amortiguación térmica), así como evaluar el máximo térmico crítico para un subconjunto de 24 especies.
4. La capacidad de amortiguación térmica y la tolerancia se influenciaron por la familia, la longitud del ala y el colour del ala; con Pieridae y mariposas grandes o de colour más oscuro teniendo la capacidad de amortiguación térmica más fuerte, pero Hesperiidae, mariposas pequeñas y de colour más oscuro tolerando las temperaturas más altas.
5. Identificamos una relación entre la capacidad de amortiguación térmica y la tolerancia fisiológica, en la que las especies con mayores capacidades de amortiguación térmica tenían una menor tolerancia térmica, y viceversa. Esta interacción implica que las especies con temperaturas corporales más estables en el campo pueden ser más vulnerables a los aumentos en las temperaturas ambientales, por ejemplo, las olas de calor asociadas con el cambio climático actual.
6. Nuestra investigación demuestra que las especies tropicales emplean diversas estrategias de termorregulación, las cuales también se reflejan en su sensibilidad a las temperaturas extremas.

## 1 INTRODUCTION

Climate change poses a severe threat to many taxa, with increases in mean temperature in many regions disrupting growth (Maino et al., 2016), behaviour and survival of individuals (Kingsolver et al., 2013), and synchronicity of ecological relationships (Cornelissen, 2011). Climate change is also increasing the frequency and intensity of extreme weather events (Jentsch et al., 2007). This puts stress on species unable to cope with short periods of extremely high temperatures or extended droughts, and has knock‐on effects on populations and wider ecological interactions. It is therefore important to understand both the impacts of changes in mean temperature and temperature extremes on animals.

Species that are unable to move to track temperature change must overcome changes in mean and extreme temperature within their local area, especially where conditions are altered outside of their preferred temperature range. Species can maintain their body temperature within tolerable limits across a range of air temperatures by using behavioural mechanisms, such as movement between suitable microhabitats (Duffy et al., 2015; thermal buffering ability). However, such strategies may not be sufficient to maintain body temperatures within tolerable limits during extreme high temperature events (De Palma et al., 2017). In such cases, species must rely on physiological processes to tolerate high temperatures, if only for relatively short periods of time (Angilletta Jr, 2009; thermal tolerance). These aspects of thermal adaptation, temperature avoidance or tolerance, may interact; behavioural adaptations can help species overcome changes in ambient conditions by enabling them to seek suitable microclimates, but may have a detrimental effect on the evolution of thermal tolerance by reducing exposure to non‐optimal temperatures, and therefore weakening the selective pressure needed to adapt (Buckley et al., 2015; Huey et al., 2003). Similarly, species that have high tolerance to high temperatures may not evolve an ability to behaviourally avoid high temperatures due to weak selective pressure, ultimately leaving them vulnerable to temperature extremes outside of their tolerance. This implies there may be an interaction between these two types of thermal adaptation. For example, under climate change, species with a strong ability to avoid non‐tolerable temperatures (such as having a high thermal buffering ability) may initially show limited responses to minor changes in ambient conditions, as microclimates can continue to act as viable thermoregulatory resources. However should temperatures continue to rise and cooler microclimates be lost, or should an extreme high‐temperature event occur, these species may be severely affected, due to a lack of long‐term selective pressure to tolerate high temperatures in these species. If this were the case, species with high thermal buffering abilities may first show limited responses to warming, but should temperatures continue to rise or thermoregulatory mechanisms break down, these species could show dramatic and rapid declines. This trade‐off may be particularly severe in the tropics, where species tend to have narrower climate niches and lower ability to disperse to climatically suitable areas than their temperate counterparts (Grinder & Wiens, 2022), and where habitat degradation, and therefore loss of suitable microclimates, is severe (Senior et al., 2019).

Butterflies are an ecologically sensitive group, showing marked responses to environmental change. Body temperature is a key determinant for many processes relevant for butterfly fitness, including flight ability (Merckx et al., 2006), reproductive behaviour (McDonald & Nijhout, 2000) and fecundity (Karlsson & Wiklund, 2005). However, excessive heat above thermal tolerances can cause irreversible protein denaturation, disturb ionic regulation and result in death (González‐Tokman et al., 2020; Heath et al., 1971). There is, therefore, a strong selective pressure for butterflies to maintain their body temperature within tolerable ranges under variable ambient conditions (thermal buffering ability, see Bladon et al., 2020). This can be done in a variety of ways, including behavioural thermoregulation (such as altering wing position relative to the sun), selecting favourable microclimates (Clench, 1966; Montejo‐Kovacevich et al., 2020) or by evolving morphological traits, such as colours or sizes, that confer thermal benefits (Wenda et al., 2021). Species with strong thermal buffering ability are likely to be at a selective advantage over species with poor thermal buffering ability, as they are able to maintain their body temperature within a tolerable range across a variety of air temperatures, meaning they are able to efficiently warm up in cool air temperatures and cool down in warm air temperatures. However, where extreme temperatures rise beyond behavioural thermoregulation capacity, and there are not suitable cool microclimates for butterflies to exploit, species must instead rely on physiological mechanisms, such as the production of heat shock proteins, to tolerate high temperature (González‐Tokman et al., 2020).

The thermal buffering ability of temperate butterflies is influenced by taxonomic family and wing length, with Pieridae and larger species tending to have better thermal buffering ability than other butterfly families and small species (Bladon et al., 2020). Large insects generally experience smaller fluctuations in body temperature than small insects (Gilchrist, 1990; Kemp & Krockenberger, 2004), and so large size should result in a higher thermal buffering ability. In cool conditions, large‐winged butterflies may also be able to use the large surface areas of their wings to absorb heat, enabling rapid warming and activity. Additionally, large wings would increase the mobility of butterflies, enabling them to access a greater variety of microclimates over a wider area than smaller butterflies (Sekar, 2012). Bladon et al. (2020) also found some evidence of wing colour influencing thermal buffering abilities of temperate species; however, this was attributed to the distribution of colours across families. Colour is known to influence heating rates in butterflies, with previous studies finding that dark butterflies tended to experience more rapid changes in body temperature than paler butterflies (Watt, 1968). These rapid fluctuations would enable darker butterflies to warm up faster in cool conditions, and to radiate heat and cool‐down more rapidly in warm conditions. As such, morphological traits may confer benefits to the thermal buffering ability of species.

Similar aspects of butterfly morphology that affect thermal buffering have also been found to influence thermal tolerance (e.g. a positive relationship between thermal tolerance and body mass in *Bicyclus anynana* Klockmann et al., 2017). Should similar traits influence both thermal buffering ability and tolerance, it is possible that traits may have synergistic or antagonistic effects on these two forms of thermal adaptation. For example, large *B. anynana* individuals have higher thermal tolerance than small individuals, and large body size may also result in reduced fluctuations in body temperature, implying that larger individuals may have both a greater capacity to maintain body temperature within tolerable limits, and higher thermal buffering ability. There is currently limited information on how tropical butterflies are responding to changes in ambient temperatures, and no work has been carried out to quantify thermal buffering ability and compare this to thermal tolerance for tropical butterflies (Dongmo et al., 2021; Fischer et al., 2014), despite the majority of butterfly species being found in the tropics (Bonebrake et al., 2010).

In this paper, we address the following questions.

1. What is the range of species‐specific thermal buffering abilities across a community of tropical butterfly species, and is this influenced by family, size, or colour?

We hypothesise that thermal buffering ability will be influenced by taxonomy and size, as found by Bladon et al. (2020), with butterflies in the family Pieridae, and larger butterflies, having stronger thermal buffering abilities than other families and smaller butterflies. We predict that darker wing colour will positively influence thermal buffering ability (Berthier, 2005; Watt, 1968), whereby absorption of solar radiation is influenced by melanin in a butterfly's wing, and darker butterflies are capable of changing their body temperature at increased rates compared to paler butterflies under a given level of solar radiation. In this study, we use tropical butterflies from a single region (Panama) so that the macro‐scale environmental conditions experienced do not differ substantially between species, which could affect our results.

2 What is the range of species‐specific thermal tolerance (critical thermal maxima) across a community of tropical butterfly species, and is this influenced by family, size or colour?

We predict that thermal tolerance will be influenced by taxonomy, as previous studies have found that physiological responses to high temperatures (e.g. heat shock proteins) are phylogenetically conserved (González‐Tokman et al., 2020). We also predict that thermal tolerance will be higher in larger species, as in other insects (Baudier et al., 2015). We predict that thermal tolerance will be independent of colour, as we could find limited evidence of a link between colour and temperature tolerance in the literature.

3 Is there a relationship between species‐specific thermal buffering ability and thermal tolerance across a community of tropical butterfly species?

We predict that thermal buffering ability will have a negative relationship with thermal tolerance, either due to species with strong thermal buffering abilities rarely experiencing high body temperatures and therefore experiencing weak selection to increase thermal tolerance, or because species with high thermal tolerance experience weak selection to improve thermal buffering ability.

## 2 MATERIALS AND METHODS

### 2.1 Study sites

Butterflies were sampled from multiple habitats in Panama from February 2020 to March 2022, across both wet and dry seasons. Data were collected in multiple locations: Gamboa (lowland managed urban green spaces) [9°6′59.13″N, 79°41′47.41″W] (elevation = 28 m), “Pipeline road” in Soberanía National Park (secondary semi‐deciduous lowland tropical wet forest) [9°7′39.04″N, 79°42′17.80″W] (elevation = 92 m), Campana in Capira District (pre‐montane wet encroaching scrub and secondary forest) [8°40′54.97″N, 79°55′25.08″W] (elevation = 327 m), Sajalices in Chame District (lowland tropical wet encroaching scrub and secondary forest) [8°40′53.55″N, 79°51′57.90″W] (elevation = 150 m), El Valle (lowland tropical wet encroaching scrub) [8°37′04.7″N, 80°0656.5″W] (elevation = 674 m), Mount Totumas (lower mountain rainforest and management agroforestry) [8°52′58.6″N, 82°41′01.3″W] (elevation = 1877 m) and San Lorenzo National Park (secondary lowland tropical wet forest) [9°14′49.2″N, 79°58′44.2″W] (elevation = 185 m). This range of sites allowed the collection of a wide variety of species across a range of air temperatures (minimum = 17.4°C, mean = 28.5°C, maximum = 39.7°C). Butterflies were identified to species level using identification guides and with the help of a local expert (ACZ). The only exception was *Calephelis* spp., which due to their complex taxonomy, were identified only to the genus level and treated as a single species during analyses.

### 2.2 Thermal buffering ability

Surveys were undertaken in all weather conditions except rain, between 07:30 and 16:00 h, and we attempted to capture any butterflies seen. Butterflies were caught in hand nets without chasing (to avoid raising butterfly body temperature). Immediately after capture, butterfly body temperature was recorded using a thermocouple with a handheld indicator (Tecpel Thermometer 305B, Tecpel Co. Ltd.), by gently pressing the probe through the net against the butterfly's thorax, without handling or touching the butterfly. Body temperature was recorded within 10 s of capture, followed by air temperature, taken with the thermocouple held at waist height in the shade. We then identified individual butterflies to species, and recorded wing length (with callipers, from the joint in the thorax to the tip of the forewing) and wing colour (ranked from: 1, almost white; 2, yellow‐green; 3, orange; 4; orange‐brown or blue; 5, brown; to 6, almost black; as established by Bladon et al., 2020). In species with multiple colours, colour values were averaged (for example, an equally black and white butterfly species would have the values for black (6) and white (1) averaged (giving 3.5)). Butterflies were marked and retained in a small cage until the end of the survey (up to a maximum of 6 h, in shade with access to water and sugar solution) to prevent re‐recording the same individuals, before being released.

### 2.3 Thermal tolerance

From January to March 2022, a subset of butterflies, captured to record their thermal buffering ability, were used for thermal tolerance experiments. Species (*n* = 24) were chosen based on high abundance. The selected individuals were retained in glassine envelopes with moistened cotton and kept outdoors in the shade at ambient temperature before measurement of thermal tolerance (within 6 h of capture). To measure critical thermal maximum (CT_max_), butterflies were placed individually into six glass jars with moistened filter paper (to prevent dehydration) in a water bath (Huber CC‐K20 with Pilot ONE, Huber Kältemaschinenbau AG) at 28°C for 5 min to acclimatise. This starting temperature was chosen as it was the average ambient air temperature recorded during capture across all butterflies. A thermocouple with a hand‐held indicator (Tecpel Thermometer 305B, Tecpel Co. Ltd.) was placed into a control jar to monitor and record in‐jar temperatures. After acclimatisation, the water bath was set to ramp up temperature steadily, at a rate of 0.5°C/min to a maximum of 70°C. By maintaining high humidity throughout the experiment and ramping temperature at an ecologically relevant rate (Terblanche et al., 2007), we aimed to simulate features of climate change in the tropics, for example a high temperature weather event, where temperature increases and humidity remains high. During the experiment, water bath internal temperatures (recorded using the water bath internal thermometer) and actual in‐jar temperatures (recorded using the thermocouple) were recorded every 5 min to ensure the set ramping rate was achieved. To prevent inter‐run differences affecting results, no more than three individuals of a single species were placed into a single run. The temperature at which each butterfly lost motor control (“knockdown”, assessed as the temperature at which the butterfly fell down and, after being poked, did not right itself) and time to knockdown were recorded (Huey et al., 1992). Ambient laboratory temperatures during the experiments ranged from 23 to 25°C. Before being placed in the water bath, wing length (measured with callipers) (Ribeiro et al., 2012) and condition (on a scale of 1–5, following Bladon et al., 2020, where 1 is perfect, 2 is scale loss but no physical damage to wings, 3 is heavy scale loss and/or light damage to wing edges, 4 is damage to multiple (but not all) wings, and 5 is significant damage on all wings) of each butterfly was recorded again. Only butterflies of conditions 1–3 were used (assessed beforehand) to prevent senescence or poor condition affecting the results. Exposure duration (including starting temperature and rate of temperature change) is known to influence critical thermal limits recorded (Terblanche et al., 2007). As the butterflies were wild‐caught, temperature variation experienced throughout the life cycle, and therefore their thermal history, may have influenced our results (Kellermann et al., 2017). However, as all individuals were randomly caught in the same season of the same year for this experiment, this effect is likely to be minimal.

### 2.4 Data processing and statistical analyses

#### 2.4.1 Data analyses: Thermal buffering ability

All analyses were conducted in R version 3.6.1 (R Core Team, 2017, http://www.r‐project.org). Plots were produced with the ‘interactions’ (Long, 2019) and ggplot2 R packages (Wickham, 2016). A total of 54 species from six butterfly families were used for analysis; Hesperiidae (11 species, 219 individuals), Lycaenidae (3 species, 67 individuals), Nymphalidae (26 species, 727 individuals), Papilionidae (3 species, 53 individuals), Pieridae (8 species, 209 individuals) and Riodinidae (3 species, 59 individuals). We excluded species which had fewer than 10 recordings over less than 5°C air temperature range, as we assessed this as insufficient to determine thermal buffering ability (Bladon et al., 2020). To test whether taxonomy or species traits were correlated (family, wing length and wing colour), pairwise one‐way ANOVA tests were used. We did not find evidence to suggest strong correlations between explanatory variables, however where relationships were detected, we considered these relationships during interpretation (Appendix, Supplementary Methods 1).

We fitted linear regression models of body temperature against air temperature for each species. The slope of this regression was used to estimate the ability of each species to “buffer” its body temperature in response to varying air temperature (i.e. its ability to maintain a stable body temperature across a wide range of air temperatures). To aid interpretation, slopes were subtracted from one (hereafter referred to as the buffering estimate), so that a small value indicated a weak thermal buffering ability and a large value indicated a strong thermal buffering ability (following Bladon et al., 2020).

To investigate the effect of family and species' traits on the thermal buffering ability of individual species, we fitted a mixed effect model with body temperature as the response variable, and air temperature, family, wing length, wing colour and two‐way interactions between air temperature and each of the other variables as explanatory variables. Species was included as a random effect to control for inter‐specific differences. The assumptions of mixed effect models were checked and met before fitting using the sjplot package (Lüdecke, 2021). Model selection was conducted through backwards stepwise selection, to avoid suppressor effects, using the lmertest package (Kuznetsova et al., 2017), where non‐significant terms were removed until a minimal model was achieved in which all remaining terms were significant. The retention of a two‐way interaction between air temperature and a trait in the optimal model indicates that the trait is important in explaining thermal buffering ability.

#### 2.4.2 Data analyses: Thermal tolerance

We included 24 species, for which sample sizes ranged from 18 to 24 individuals, in the analyses. We ensured at least 18 individuals were tested, as this was the highest number we could record per species in the available time, and ensured there was sufficient replication to resolve thermal tolerance. Five of the six butterfly families were represented, Hesperiidae (7 species, 143 individuals), Lycaenidae (3 species, 61 individuals), Nymphalidae (9 species, 186 individuals), Pieridae (3 species, 67 individuals) and Riodinidae (2 species, 42 individuals). There were no species of Papilionidae with sufficient numbers to include. The temperature at which 50% of individuals of each species were knocked down (hereafter, LD50) was calculated using the survival package (Therneau, 2022). Differences between water bath runs were checked before analyses and found to not differ (Appendix, Supplementary Methods 2).

To investigate whether family, wing length, wing colour (using the same 1–6 scale described above) or thermal buffering ability affected thermal tolerance, survival probabilities were assessed with the Kaplan–Meier method using the survival package (Therneau, 2022). Differences between survival curves for family, wing length, wing colour and thermal buffering ability were specifically tested with log‐rank tests in individual models. Plots were produced using the survminer package (Kassambara et al., 2021).

## 3 RESULTS

### 3.1 Thermal buffering ability

A total of 1334 butterflies were included in analyses of thermal buffering ability, covering 54 species from six butterfly families. Air temperatures at which butterflies were captured ranged from 17.4 to 39.7°C. Species‐specific buffering estimates ranged from −0.32 (*Hemiargus hanno*) to 1.00 (*Phoebis argante*) (Table S1, Figures S1–S6).

**FIGURE 1:**
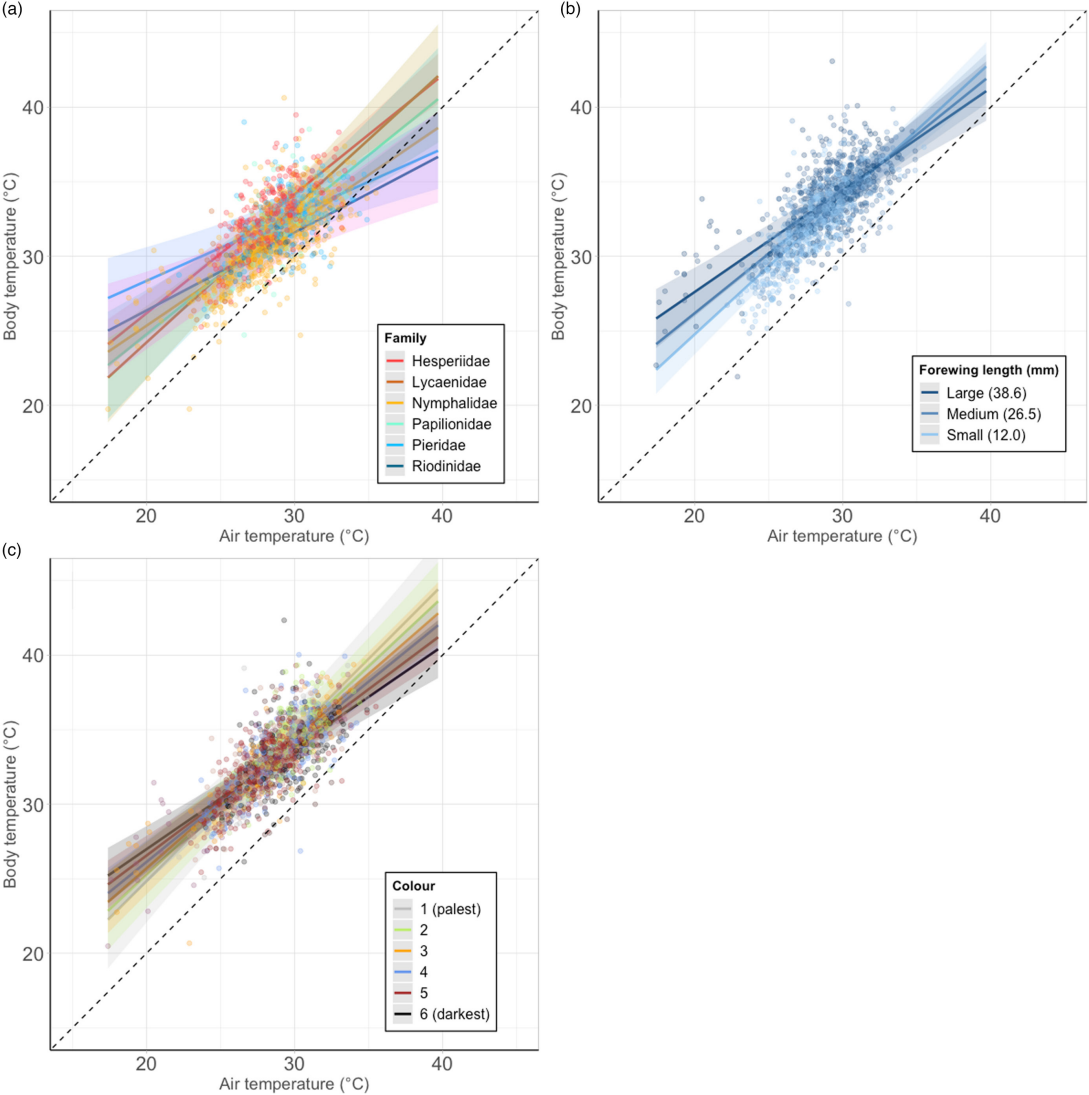
Differences in the relationship between air temperature (°C) and body temperature (°C) (thermal buffering ability) between (a) families, (b) forewing lengths (in mm, modelled as a continuous variable but split into three groups for plotting: medium (mean wing length), large (one standard deviation above the mean) and small [one standard deviation below the mean]), and (c) wing colours (on a scale from 1 (almost completely white) to 6 (almost completely black), assessed using established methods (Bladon et al., 2020; colour of points and lines represent actual butterfly wing colours those categories correspond to). Lines represent predicted values restricted to the range of temperatures observed. Shaded areas show 95% confidence intervals. Points represent partial residuals (observed data points of individual butterflies with the effects of the other variables accounted for). Dashed lines show a 1:1 relationship between air and butterfly temperature to aid interpretation.

Across the community of tropical butterflies, family, wing length and wing colour all affected thermal buffering ability (Tables S3 and S4). The families differed in thermal buffering abilities (*χ* ^2^_1,5_ = 12.87, *p* = 0.025), with Pieridae having the highest buffering estimate (mean = 0.41, range: 0.07–1.00), followed by Riodinidae (mean = 0.38, range: 0.31–0.45), Papilionidae (mean = 0.37, range: 0.18–0.55), Nymphalidae (mean = 0.33, range: −0.15–0.79), Hesperiidae (mean = 0.19, range: −0.26–0.69) and Lycaenidae (mean = −0.1, range: −0.32–0.16; Figure 1a). Larger species had higher buffering estimates than smaller species (*χ* ^2^_1_ = 16.52, *p* < 0.001; Figure 1b). Darker species had higher buffering estimates than paler species (*χ* ^2^_1_ = 4.41, *p* = 0.036; Figure 1c). However, these trends are likely to be driven by Nymphalidae, as this family encompasses the majority of variation in the traits tested.

### 3.2 Thermal tolerance

Across 24 species and five butterfly families, temperatures at which 50% of individuals were knocked down (LD50) ranged from 45.05°C (*Itaballia demophile*) to 56.80°C (*Junonia zonalis*). The range of temperatures between which 10% and 90% of individuals were knocked down varied from 2.5°C (*Dione juno*) to 15.1°C (*Spicauda procne*) between species (Table S5, Figure S7).

**FIGURE 2:**
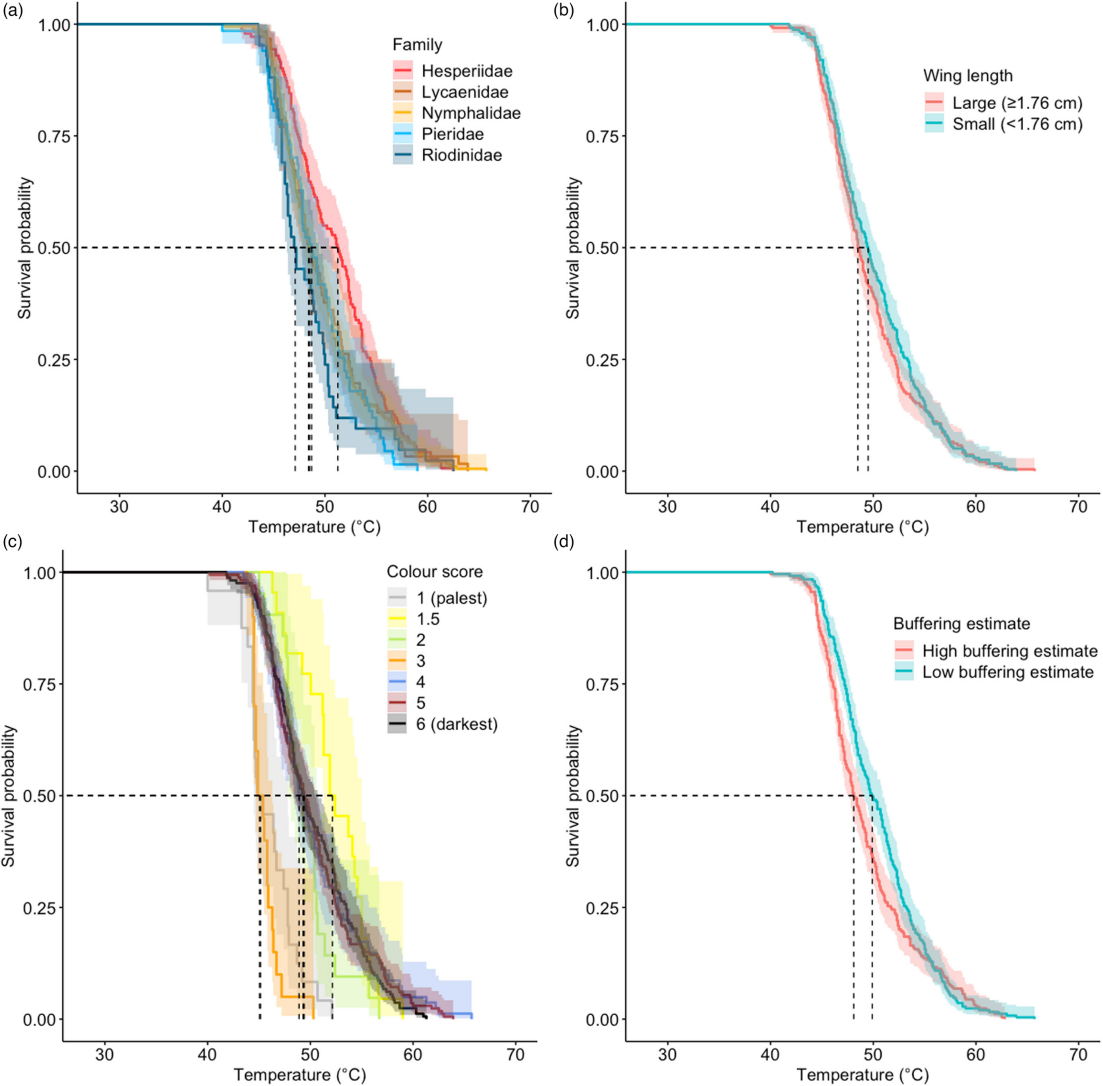
Average thermal survival curves across (a) families, (b) forewing length (modelled as a continuous variable but split into two groups for plotting: small [below median] and large [above median] [median = 17.8 mm]), (c) wing colours (on a scale from 1 (almost completely white) to 6 (almost completely black), assessed using established methods (Bladon et al., 2020) (colour of points and lines represent actual butterfly wing colours those categories correspond to) and (d) buffering abilities (modelled as a continuous variable but split into two groups for plotting: weak buffering ability [below median] and strong buffering ability [above median] [median = 0.211]). During the experiment, temperature was increased from 28°C to 70°C at 0.5°C per minute until the butterfly was knocked down (see Section 2). Solid lines represent mean survival, ribbons represent 95% confidence intervals. Dashed lines show the temperature at which 50% of individuals were knocked down (LD50) per group.

Survival curves differed between butterfly families (*χ* ^2^_1,4_ = 13.9, *p* = 0.007): Hesperiidae tolerated the highest temperatures before knock down (LD50: 51.3°C, knock down range: 11.4°C), followed by Pieridae (LD50: 48.7°C, knock down range: 10.6°C), Nymphalidae (LD50: 48.5°C, knock down range: 11.8°C) and Lycaenidae (LD50: 48.4°C, knock down range: 11.6°C), while Riodinidae had the lowest LD50 and narrowest knock down range (LD50: 47.1°C, knock down range: 8.4°C; Figure 2a).

Survival curves differed between individuals with different wing lengths (*χ* ^2^_1,212_ = 718.0, *p* < 0.001). Small butterflies tolerated higher temperatures than large butterflies (small, forewing length ≤ median (17.9 mm), LD50: 49.5°C, knock down range: 11.3°C; large, forewing length > 17.9 mm, LD50: 48.5°C, knock down range: 11.9°C; Figure 2b).

Survival curves differed between individuals with different coloured wings (*χ* ^2^_1,6_ = 116.0, *p* < 0.001). Darker butterflies tolerated higher temperatures than paler butterflies (darker butterflies (colour value >3), LD50: 49.3°C, knock down range: 11.7°C; paler butterflies (colour value ≤3), LD50: 48.7°C, knock down range: 10.6°C; Figure 2c).

Survival curves differed between individuals with different thermal buffering abilities (*χ* ^2^_1,23_ = 263.0, *p* < 0.001). Individuals with low buffering estimates were able to tolerate higher temperatures than individuals with high buffering estimates (weak thermal buffering ability ≤ median (0.21), LD50: 49.9°C, knock down range: 11.3°C; strong thermal buffering ability >0.21 LD50: 48.6°C, knock down range: 12.3°C; Figure 2d, Figure S8).

## 4 DISCUSSION

Our study identified widely differing thermal buffering abilities and tolerances, which varied systematically with family, wing length and wing colour, and interacted with each other, across a large assemblage of tropical butterflies. We found that thermal buffering ability was influenced by taxonomic family. Pieridae had the strongest thermal buffering ability and were able to behaviourally avoid high body temperatures. Contrastingly, Lycaenidae had the weakest thermal buffering ability, being particularly poor at heating up in cool weather and cooling down in hot weather. We also identified wing length and colour as traits that influence thermal buffering abilities. Overall, small, paler butterflies were particularly poor at buffering against changes in air temperature, whereas large, darker butterflies had stronger buffering abilities, being more able to maintain a relatively stable body temperature across variable air temperatures. Thermal tolerance also differed between families, species, wing lengths and colours. Generally, Hesperiidae tolerated the highest temperatures, whereas Riodinidae had the lowest thermal tolerance. Small, darker species had the highest thermal tolerance. For the first time, we also found a negative relationship between thermal buffering ability and thermal tolerance, indicating an interaction between these strategies. Our findings provide important information for predicting characteristics of species at greater risk from increases in mean and extreme temperature events in the biodiverse tropics.

### 4.1 Taxonomic group

We found that butterfly families differed in thermoregulation strategies and tolerance to high temperatures. This is in‐line with studies that have found that heat tolerance mechanisms, such as heat shock proteins, are highly phylogenetically conserved (Nguyen et al., 2016), and suggests that thermal tolerance has heritable components that could be favoured by natural selection. Pieridae had the highest thermal buffering ability of the six butterfly families, as well as relatively high thermal tolerance. Pieridae were also found to have the highest thermal buffering abilities in a similar study on temperate butterflies (Bladon et al., 2020). Pieridae generally bask to raise their body temperature (Watt, 1968), sometimes using their light wings to reflect solar energy onto their body (Shanks et al., 2015). Their generally large wings and paler colouration may allow Pieridae to reflect light more efficiently than other families, and avoid absorbing excess solar energy when not basking.

Conversely, Lycaenidae had the poorest thermal buffering ability, and low thermal tolerance. Lycaenidae is one of the most speciose butterfly families (Robbins, 1982), and commonly consists of small butterflies with bright, often iridescent, colouration. A study on a small temperate Lycaenidae found that wing colour did not strongly impact heating rates, although weight was important (De Keyser et al., 2015). This indicates that it may not be the colouration of Lycaenids that makes them poor at buffering temperature, but rather their small size. The poor thermal buffering ability and low thermal tolerance in this group implies that Lycaenidae may be disproportionately affected by changes in ambient temperatures.

Hesperiidae tolerated the highest temperatures of the five families tested, but had relatively weak thermal buffering abilities. This implies that tropical hesperiids may compensate for their poor thermal buffering ability with higher thermal tolerance. Alternatively, their high thermal tolerance may reduce selective pressure to maintain their body temperature within a narrow range. Hesperiidae include butterflies that have short wings and large stout bodies and rapid wing beats (Betts & Wootton, 1988). As wingbeat frequency is temperature dependent (Kammer, 1970), the characteristic rapid wingbeats of hesperiids may require higher thoracic temperatures for flight than other butterfly groups, and so they are more likely to experience high body temperatures and undergo selection to increase thermal tolerance, however there is limited evidence to support this (Nève & Hall, 2016). Alternatively, being large‐bodied, hesperiids may retain heat more than small‐bodied butterflies. This would result in hesperiids being less able to lose heat in warm weather, possibly resulting in them commonly experiencing high body temperatures, and therefore evolving higher temperature tolerance.

Riodinidae had the lowest thermal tolerance across the five families, but had relatively strong thermal buffering abilities. This result suggests they are able to behaviourally avoid high body temperatures, which may compensate for their low thermal tolerance. Alternatively, having low thermal tolerance, Riodinidae may be under strong selective pressure to develop mechanisms to maintain their body temperature within a relatively narrow tolerable range. Many Riodinidae frequently rest on the ventral surface of leaves which tend to be cooler than the dorsal surface (Pallas et al., 1967), and, as such, are rarely seen in direct sunlight. This behaviour may allow the most thermally‐sensitive species to persist in high temperature environments, and also offers an explanation for their strong thermal buffering abilities. This implies that tropical riodinids may be able to cope with changes in mean temperatures, but it is unclear to what extent they will be able to cope with extreme high temperature events.

### 4.2 Wing length

Large winged butterflies had stronger thermal buffering abilities than small butterflies, possibly due to a combination of behavioural and morphological adaptations, such as a greater ability to fly long distances to search for suitable microclimates across a wider area. This result aligns with a similar study on temperate butterflies (Bladon et al., 2020), implying that this is a consistent trend across regions. Previous studies have found that large insects raise and lower their body temperature at a slower rate than small insects (Kemp & Krockenberger, 2004), and tend to have more stable body temperatures (Gilchrist, 1990). This relative stability, as well as their larger wings, could enable large species to travel further and faster to find suitable microclimates, further buffering their body temperature. Large butterflies could also use their large wings to absorb solar energy more quickly, or reflect more solar radiation onto their bodies (Shanks et al., 2015), and so increase their body temperature faster when basking than small species.

We also found that large butterflies tended to have lower thermal tolerance than small butterflies. As butterfly body mass correlates with wing length (Peixoto & Benson, 2008), our finding differs from previous studies on tropical butterflies, which found thermal tolerance increased with mass (Klockmann et al., 2017; Luo et al., 2014). However, these studies tended to be based on single species, and may not reflect patterns across a community. It is possible that the negative relationship we found between wing length and thermal tolerance is related to the higher metabolic rate and oxygen demand in larger insects (Lachenicht et al., 2010). This would make large butterflies particularly sensitive to further increases in temperature, whereby their metabolism increases beyond oxygen delivery.

### 4.3 Wing colour

Darker butterflies had stronger thermal buffering abilities than paler butterflies, and darker butterfly species also could tolerate higher temperatures than paler butterflies. This is in‐line with previous evidence that dark butterflies heat up and cool down faster than pale butterflies at a given level of solar radiation (Watt, 1968), and achieve higher body temperatures than pale individuals (Dufour et al., 2018; Khazan et al., 2022). This is also in line with comparative studies across temperate latitudes (Zeuss et al., 2014), which found a higher incidence of darker species in cooler conditions, possibly also related to the advantage of darker species in being able to warm themselves in cooler conditions. Darker species may be more likely to experience high body temperatures and be adapted to cope with the predicted increases in ambient temperatures under climate change. Paler butterflies may benefit from rising temperatures in the tropics, by enabling them to gain heat and become active more quickly. However, as well as warming up slower, paler butterflies are also less able to lose heat at high ambient temperatures compared to darker butterflies (Watt, 1968), and we found them to have lower thermal tolerance than darker butterflies, putting them at an increased risk of overheating under rising temperatures.

Temporal shifts in activity may benefit some species under climate change, whereby they shift their activity to the cooler parts of the day (for example, dawn or dusk). Alternatively, species may shift spatially to compensate for changes in ambient conditions, for example occupying spaces with adequate cool microclimates during the hottest parts of the day. However, habitat loss, fragmentation, or homogenisation, all of which are particularly prevalent in the tropics, would reduce the ability of species to both find cool microclimates and to increase the severity of temperature extremes (Senior et al., 2019). Therefore, habitat loss may amplify the effects of changes in ambient temperatures, and pose a challenge to both species with poor thermal buffering abilities or low thermal tolerance.

### 4.4 The relationship between thermal buffering ability and thermal tolerance

Our survival analysis found a negative relationship between thermal buffering ability and thermal tolerance, implying that there could be a trade‐off between avoiding or tolerating high temperatures, with species favouring one of these strategies at the expense of the other. This finding may partially reflect mechanistic links between factors affecting the two methods for coping with higher temperatures. For example, smaller species were less able to buffer their body temperature, but were more able to tolerate higher temperatures. However, in the case of colouration, darker species were both better able to buffer temperature and tolerate high temperatures. An alternative interpretation is that thermal tolerance has evolved as a result of butterflies with poorer thermal buffering ability being more likely to regularly experience high body temperatures. In contrast, species with strong thermal buffering abilities may be under relatively weak selective pressure to evolve high thermal tolerance: their ability to maintain their body temperature within tolerable ranges means they rarely experience high body temperatures. Given that both average temperatures and extreme temperatures are predicted to increase with climate change, this negative relationship between buffering and tolerance may have a large negative effect on many butterfly species, as few species are likely to have both an ability to buffer against average increases and an ability to tolerate extremes.

It is important to note that the patterns we have detected in both thermal buffering ability and thermal tolerance are dependent on a wide range of butterfly families, and therefore size and colour traits included in our study. The wide range in traits we have included has the advantage of enabling us to detect patterns that would not be possible from single family studies, as trait variation would not be so high. However, our findings do come with the caveat that they are dependent on cross‐family differences in traits, and are like to be particularly driven by abundant families such as nymphalids. However, the significant additional variation not dependent on family in traits that we found, as well as contrasting family and trait effects (such as darker butterflies and the palest family, Pieridae, being better at thermal buffering), indicates that the traits we have detected are reliable indicators of thermal buffering and tolerance.

A further caveat to consider is that the data presented was collected only on the adult life stage, whereas other life stages may be more vulnerable to changing temperatures (Radchuk et al., 2013). Future studies should address this gap in our understanding of tropical butterfly responses to temperature across the life cycle.

## 5 CONCLUSIONS

Our findings have identified family, wing length and wing colour as factors influencing the ability of butterflies to cope with temperature change. These findings are strikingly similar to a study on temperate butterflies, which identified Pieridae and large butterflies as having the strongest thermal buffering abilities (Bladon et al., 2020). This implies a consistent pattern across tropical and temperate butterfly species. These findings provide important information to predict which traits, and species with these traits, may be selected for under warming temperatures in the tropics. Our results also indicate that species at risk under higher average and extreme temperature events are predictable based on traits. In particular, Lycaenidae are likely to be ‘losers’ under future climate change, and as a species‐rich family, there may be high species losses in the tropics. However, the trade‐off between thermal buffering ability and thermal tolerance implies that most species will be vulnerable to climate change to an extent, considering that both of these changes are predicted to increase in the future, and species appear to adapt to one strategy at the expense of the other. In particular, species with strong thermal buffering abilities may initially show limited responses to changes in ambient conditions, but should microclimates be lost or an extreme high temperature event were to occur, these species may show dramatic declines due to lacking the selective pressure to tolerate high temperatures. More work is needed to unpick how these two strategies interact with a species' ability to cope with temperature change.

## AUTHOR CONTRIBUTIONS

Study was designed by Esme Ashe‐Jepson, Andrew J. Bladon, Benita C. Laird‐Hopkins, Greg P.A. Lamarre, Edgar C. Turner and Yves Basset. Data were collected by Esme Ashe‐Jepson, Ana Cecilia Zamora, Alex Mcfarlane, Amanda F. Savage, Benita C. Laird‐Hopkins and Stephany Arizala Cobo. Esme Ashe‐Jepson conducted analyses and wrote the first draft of the manuscript. All authors contributed substantially to revisions.

## CONFLICT OF INTEREST STATEMENT

None to declare.

## Supporting information


**Appendix 1.** Table S1: List of 54 species, ordered alphabetically by family, with their estimated buffering ability obtained from a regression of body temperature against air temperature. Buffering ability was calculated as the slope of this regression subtracted from 1 (see Methods). Also listed are the colour values assigned to each species (on a scale of 1 (almost white) to 6 (almost black) as established by Bladon et al. 2020, see Methods), range in wing lengths of individuals sampled (forewing length in mm from the joint at the thorax to the tip), and the total number of each species recorded (sample size). Due to the complex taxonomy of the *Calephelis* genus, individuals of this group were not identified to species level.
**Table S2:** List of species with photo credits for insets used in Figs S1–S7 and Table S5.
**Table S3:** Mixed effect linear model results, with all fixed effects and interaction effects (denoted by a colon between fixed effects) listed. Significant p‐values are in bold. As all interaction terms were significant in the full model, no model selection was necessary.
**Table S4:** The intercepts and slopes (±1 standard error) for each term in the mixed effect linear model. Slope estimates for each family indicate the interaction with air temperature.
**Table S5:** List of 24 species included in thermal tolerance analyses, ordered alphabetically by family, with the temperature at which 50% of individuals were knocked down (LD50), and the difference between the temperatures at which 90% were still standing and the temperature at which 10% were still standing (knock down range). The number of individuals per species is also shown (sample size). Two species followed by * were excluded from analysis of thermal buffering ability and thermal tolerance due to insufficient thermal buffering data. For inset photo credits, see Table S2.
**Figure S1:** The relationship between body temperature (°C) and air temperature (°C) for 11 species of Hesperiidae. Points show individual butterflies. Red lines show the linear regression between air and body temperature. Shaded areas show 95% confidence intervals. Black lines show a 1:1 relationship to aid visual comparison between species. For inset photo credits, see Table S2.
**Figure S2:** The relationship between body temperature (°C) and air temperature (°C) for three species of Lycaenidae. Points show individual butterflies. Red lines show the linear relationship between air and body temperature. Shaded areas show 95% confidence intervals. Black lines show a 1:1 relationship to aid visual comparison between species. For inset photo credits, see Table S2.
**Figure S3:** The relationship between body temperature (°C) and air temperature (°C) for 26 species of Nymphalidae. Points show individual butterflies. Red lines show the linear relationship between air and body temperature. Shaded areas show 95% confidence intervals. Black lines show a 1:1 relationship to aid visual comparison between species. For inset photo credits, see Table S2.
**Figure S4:** The relationship between body temperature (°C) and air temperature (°C) for three species of Papilionidae. Points show individual butterflies. Red lines show the linear relationship between air and body temperature. Shaded areas show 95% confidence intervals. Black lines show a 1:1 relationship to aid visual comparison between species. For inset photo credits, see Table S2.
**Figure S5:** The relationship between body temperature (°C) and air temperature (°C) for eight species of Pieridae. Points show individual butterflies. Red lines show the linear relationship between air and body temperature. Shaded areas show 95% confidence intervals. Black lines show a 1:1 relationship to aid visual comparison between species. For inset photo credits, see Table S2.
**Figure S6:** The relationship between body temperature (°C) and air temperature (°C) for three species of Riodinidae. Points show individual butterflies. Red lines show the linear relationship between air and body temperature. Shaded areas show 95% confidence intervals. Black lines show a 1:1 relationship to aid visual comparison between species. For inset photo credits, see Table S2.
**Figure S7:** Average thermal survival curves for 24 species. Temperature was increased from 28°C to 70°C at 0.5°C per minute until the butterfly was knocked down (see Methods). Red solid lines show mean survival, ribbons represent 95% confidence intervals. Dashed lines show the temperature at which 50% of individuals were knocked down (LD50) per species. Species are ordered alphabetically by family. For inset photo credits, see Table S2.
**Figure S8.** The relationship between species‐specific buffering estimate and thermal tolerance of 22 species for which both values were calculated (two species that had their thermal tolerance calculated did not have sufficient data to calculate thermal buffering ability and were excluded from this analysis). Points represent the average temperature at which 50% of individuals were knocked down (LD50). Solid lines represent the range within species between which 10% and 90% of individuals were knocked down (the difference between which is the knock down range). Dotted lines represent the total temperature range within species from the first to last knocked down individual. Points are coloured by family, and have been jittered to make overlaying points visible.

## Data Availability

Data available from the Apollo—University of Cambridge Repository https://doi.org/10.17863/CAM.97060 (Ashe‐Jepson et al., 2023).
