# Supplementary material for: Tropical butterflies use thermal buffering and thermal tolerance as alternative strategies to cope with temperature increase
Source: J Anim Ecol. 2023 Jul 12;92(9):1759–70. doi: 10.1111/1365-2656.13970 (PMC10953451; doi:10.1111/1365-2656.13970)
Supplement: Supplementary file 1 — Appendix 1. Table S1: List of 54 species, ordered alphabetically by family, with their estimated buffering ability obtained from a regression of body temperature against air temperature. Buffering ability was calculated as the slope of this regression subtracted from 1 (see Methods). Also listed are the colour values assigned to each species (on a scale of 1 (almost white) to 6 (almost black) as established by Bladon et al. 2020, see Methods), range in wing lengths of individuals sampled (forewing length in mm from the joint at the thorax to the tip), and the total number of each species recorded (sample size). Due to the complex taxonomy of the Calephelis genus, individuals of this group were not identified to species level. Table S2: List of species with photo credits for insets used in Figs S1–S7 and Table S5. Table S3: Mixed effect linear model results, with all fixed effects and interaction effects (denoted by a colon between fixed effects) listed. Significant p‐values are in bold. As all interaction terms were significant in the full model, no model selection was necessary. Table S4: The intercepts and slopes (±1 standard error) for each term in the mixed effect linear model. Slope estimates for each family indicate the interaction with air temperature. Table S5: List of 24 species included in thermal tolerance analyses, ordered alphabetically by family, with the temperature at which 50% of individuals were knocked down (LD50), and the difference between the temperatures at which 90% were still standing and the temperature at which 10% were still standing (knock down range). The number of individuals per species is also shown (sample size). Two species followed by * were excluded from analysis of thermal buffering ability and thermal tolerance due to insufficient thermal buffering data. For inset photo credits, see Table S2. Figure S1: The relationship between body temperature (°C) and air temperature (°C) for 11 species of Hesperiidae. Points show individual [file JANE-92-1759-s001.docx]

**Appendix 1**

*Supplementary Methods: 1*

Across species, traits were associated with the butterfly families. Families differed in the frequency of different colour categories, with Pieridae being paler than the other families (R^2^ = 0.67, F_1,5_ = 517.30, p < 0.001). Families also differed in wing length, with Papilionidae being larger than the other families (R^2^ = 0.37, F_1,5_ = 145.30, p < 0.001). Wing length and colour were not correlated (cor < 0.01, F = 0.02, p = 0.896). All pairs of variables had correlation coefficients <0.7 and all were therefore retained in the model (Dormann *et al.* 2013), however their association was considered during interpretation.

*Supplementary Methods: 2*

To check that the temperature ramp on each run of the water bath had proceeded in a similar way, we fitted a linear regression, with in-jar temperature as the dependent variable, and water bath set temperature and run number as explanatory variables. An interaction effect was included between internal water bath temperature and run number to test for differences between runs. There was no significant difference in ramping rate between water bath runs (F < 0.001, d.f. = 3,1213, p = 0.94). The average ramp rate across all water bath runs was 0.414°C per minute. We therefore concluded that all runs were similar and did not include this as a factor in subsequent analyses.

*Supplementary Figures and Tables*

Table S1: List of 54 species, ordered alphabetically by family, with their estimated buffering ability obtained from a regression of body temperature against air temperature. Buffering ability was calculated as the slope of this regression subtracted from 1 (see Methods). Also listed are the colour values assigned to each species (on a scale of 1 (almost white) to 6 (almost black) as established by Bladon et al. 2020, see Methods), range in wing lengths of individuals sampled (forewing length in mm from the joint at the thorax to the tip), and the total number of each species recorded (sample size). Due to the complex taxonomy of the *Calephelis* genus, individuals of this group were not identified to species level.

| **Family** | **Species** | **Buffering estimate** | **Colour** | **Wing length range (mm)** | **Sample size** |
| --- | --- | --- | --- | --- | --- |
| Hesperiidae | *Aides dysoni* | -0.2110 | 5 | 18.9 - 27.9 | 14 |
| Hesperiidae | *Burnsius orcus* | 0.2303 | 3.5 | 12.9 - 16.7 | 26 |
| Hesperiidae | *Cecropterus dorantes* | 0.0641 | 5 | 20.4 - 35.5 | 13 |
| Hesperiidae | *Hylephila phyleus* | 0.6856 | 4 | 12.1 - 17.3 | 16 |
| Hesperiidae | *Pompeius pompeius* | 0.1704 | 5 | 12.1 - 16.8 | 36 |
| Hesperiidae | *Spicauda procne* | 0.4225 | 5 | 19.2 - 25.3 | 29 |
| Hesperiidae | *Spicauda simplicius* | -0.2550 | 5 | 18.4 - 25.5 | 27 |
| Hesperiidae | *Staphylus ascalaphus* | 0.1017 | 5 | 10.4 - 17.8 | 15 |
| Hesperiidae | *Staphylus azteca* | 0.4130 | 5 | 11.3 - 14.3 | 10 |
| Hesperiidae | *Staphylus vulgata* | 0.3604 | 5 | 11.1 - 17.0 | 12 |
| Hesperiidae | *Vehilius stictomenes* | 0.0895 | 5 | 9.0 - 13.9 | 21 |
| Lycaenidae | *Arawacus togarna* | 0.1565 | 3.5 | 12.3 - 16.2 | 13 |
| Lycaenidae | *Cupido comyntas* | -0.0919 | 4 | 8.3 - 11.2 | 12 |
| Lycaenidae | *Hemiargus hanno* | -0.3167 | 4 | 6.8 - 11.8 | 42 |
| Nymphalidae | *Adelpha cytherea* | 0.3636 | 4 | 20.5 - 28.2 | 24 |
| Nymphalidae | *Anartia fatima* | 0.3397 | 5.5 | 25.5 - 33.7 | 63 |
| Nymphalidae | *Anartia jatrophae* | -0.1132 | 3.5 | 24.3 - 33.6 | 27 |
| Nymphalidae | *Anthanassa tulcis* | 0.2978 | 4 | 13.6 - 20.0 | 30 |
| Nymphalidae | *Chlosyne lacinia* | 0.0911 | 4 | 17.8 - 26.8 | 16 |
| Nymphalidae | *Cissia pompilia* | -0.0986 | 5 | 15.9 - 21.4 | 18 |
| Nymphalidae | *Cissia terrestris* | 0.4596 | 5 | 14.0 - 18.4 | 13 |
| Nymphalidae | *Danaus plexippus* | 0.5468 | 4.5 | 42.0 - 51.2 | 22 |
| Nymphalidae | *Dione juno* | 0.3656 | 3 | 31.7 - 43.8 | 20 |
| Nymphalidae | *Dione moneta* | 0.6758 | 3 | 34.1 - 46.8 | 12 |
| Nymphalidae | *Dryas iulia* | 0.2855 | 3 | 35.5 - 46.9 | 36 |
| Nymphalidae | *Dynamine paulina* | 0.1407 | 4 | 17.8 - 31.1 | 17 |
| Nymphalidae | *Heliconius doris* | 0.4079 | 5.5 | 33.5 - 45.4 | 42 |
| Nymphalidae | *Heliconius erato* | 0.6261 | 6 | 23.7 - 39.4 | 65 |
| Nymphalidae | *Heliconius hecale* | 0.5327 | 4.5 | 36.2 - 49.8 | 44 |
| Nymphalidae | *Heliconius sara* | 0.6011 | 5.5 | 22.6 - 37.6 | 35 |
| Nymphalidae | *Hermeuptychia hermes* | -0.0550 | 5 | 13.3 - 25.5 | 47 |
| Nymphalidae | *Janatella leucodesma* | 0.3102 | 3.5 | 13.0 - 22.5 | 35 |
| Nymphalidae | *Junonia zonalis* | 0.4977 | 4 | 23.1 - 29.7 | 50 |
| Nymphalidae | *Magneuptychia libye* | 0.1617 | 4 | 22.4 - 27.0 | 13 |
| Nymphalidae | *Marpesia chiron* | 0.7864 | 5 | 25.0 - 40.7 | 10 |
| Nymphalidae | *Melinaea idae* | 0.5114 | 5 | 35.5 - 40.7 | 10 |
| Nymphalidae | *Morpho helenor* | 0.2964 | 3 | 50.2 - 70.0 | 25 |
| Nymphalidae | *Morpho menelaus* | 0.6480 | 3 | 50.9 - 76.0 | 13 |
| Nymphalidae | *Nica flavilla* | -0.1490 | 4 | 14.5 - 25.8 | 28 |
| Nymphalidae | *Pareuptychia ocirrhoe* | 0.0300 | 5 | 17.1 - 21.0 | 12 |
| Papilionidae | *Battus polydamas* | 0.1833 | 6 | 33.9 - 52.2 | 15 |
| Papilionidae | *Parides eurimedes* | 0.5526 | 6 | 31.0 - 48.5 | 14 |
| Papilionidae | *Parides sesostris* | 0.3674 | 6 | 30.0 - 47.7 | 24 |
| Pieridae | *Aphrissa statira* | 0.4366 | 2 | 24.9 - 35.6 | 24 |
| Pieridae | *Eurema albula* | 0.3999 | 1 | 8.6 - 25.2 | 22 |
| Pieridae | *Eurema daira* | 0.1655 | 1.5 | 13.8 - 19.5 | 28 |
| Pieridae | *Itaballia demophile* | 0.2112 | 1 | 22.4 - 31.7 | 34 |
| Pieridae | *Itaballia pandosia* | 0.0722 | 1 | 17.9 - 24.8 | 13 |
| Pieridae | *Phoebis argante* | 1.0045 | 2 | 25.6 - 38.3 | 39 |
| Pieridae | *Phoebis philea* | 0.3374 | 2 | 31.4 - 45.3 | 12 |
| Pieridae | *Phoebis sennae* | 0.6610 | 2 | 29.6 - 38.7 | 37 |
| Riodinidae | *Calephelis* sp. | 0.3129 | 4 | 9.3 - 13.2 | 27 |
| Riodinidae | *Detritivora hermodora* | 0.4516 | 4 | 9.6 - 14.5 | 14 |
| Riodinidae | *Juditha caucana* | 0.3624 | 3.5 | 13.3 - 18.0 | 18 |


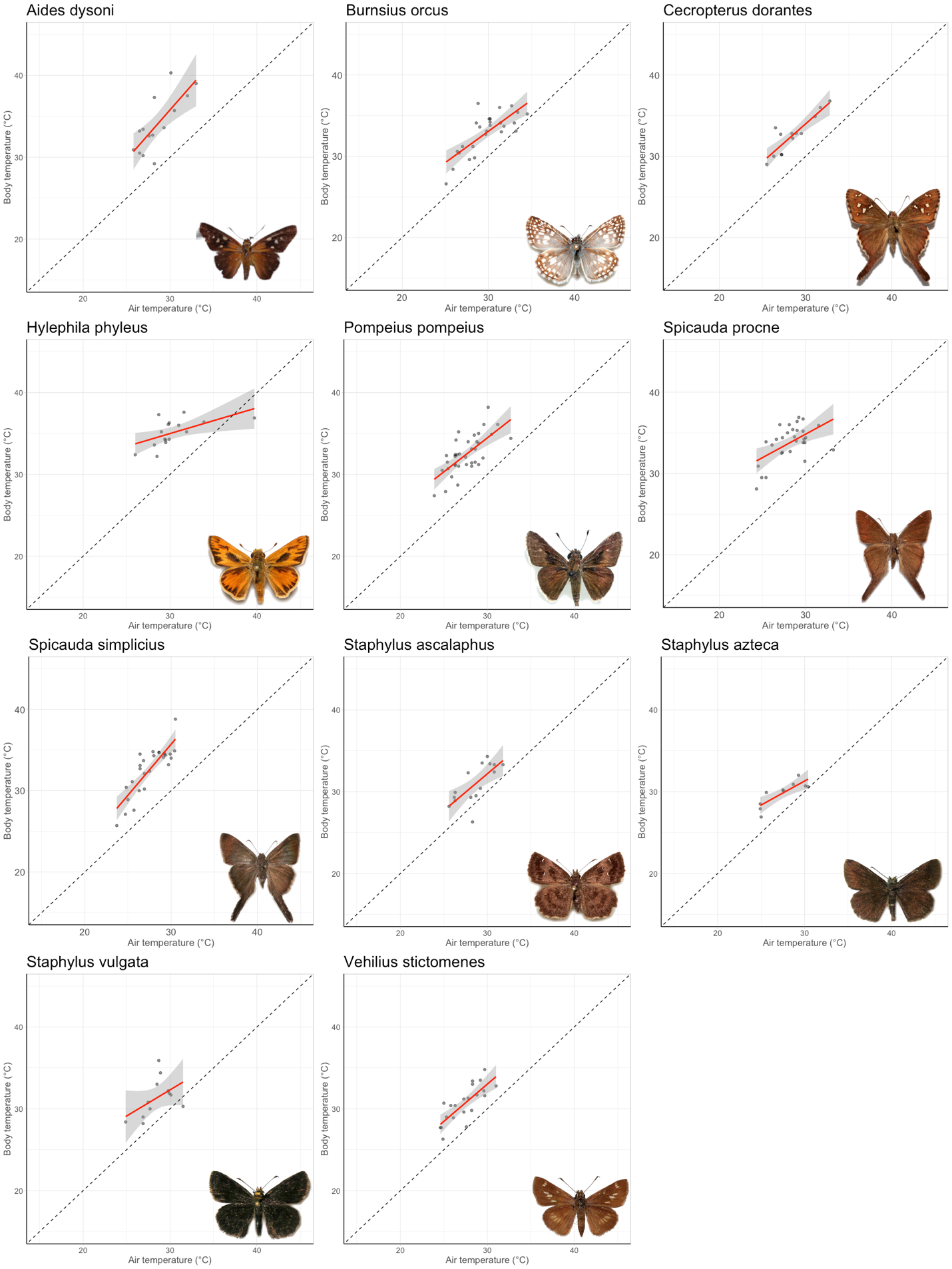


Figure S1: The relationship between body temperature (°C) and air temperature (°C) for 11 species of Hesperiidae. Points show individual butterflies. Red lines show the linear regression between air and body temperature. Shaded areas show 95% confidence intervals. Black lines show a 1:1 relationship to aid visual comparison between species. For inset photo credits, see Table S2.


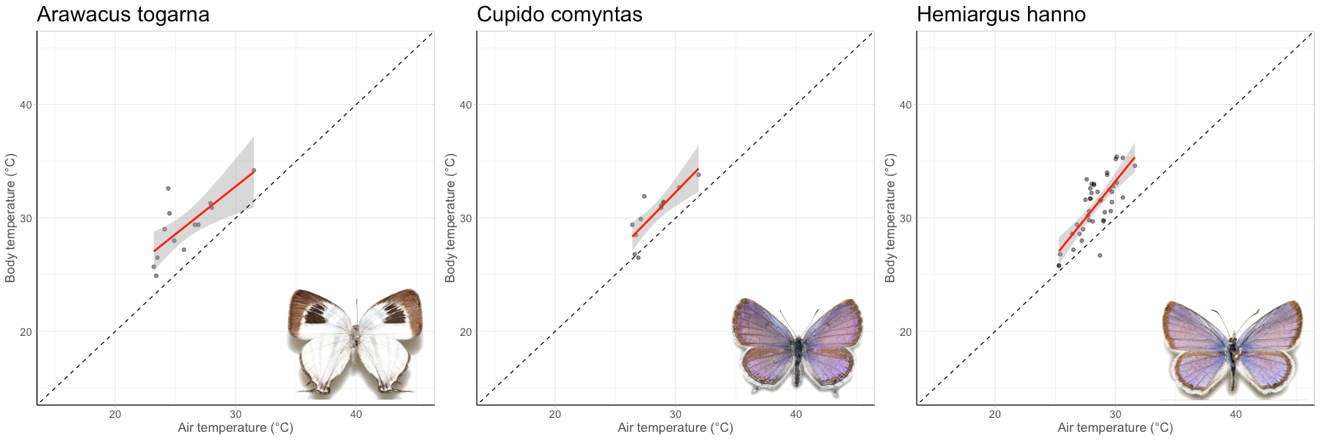


Figure S2: The relationship between body temperature (°C) and air temperature (°C) for three species of Lycaenidae. Points show individual butterflies. Red lines show the linear relationship between air and body temperature. Shaded areas show 95% confidence intervals. Black lines show a 1:1 relationship to aid visual comparison between species. For inset photo credits, see Table S2.


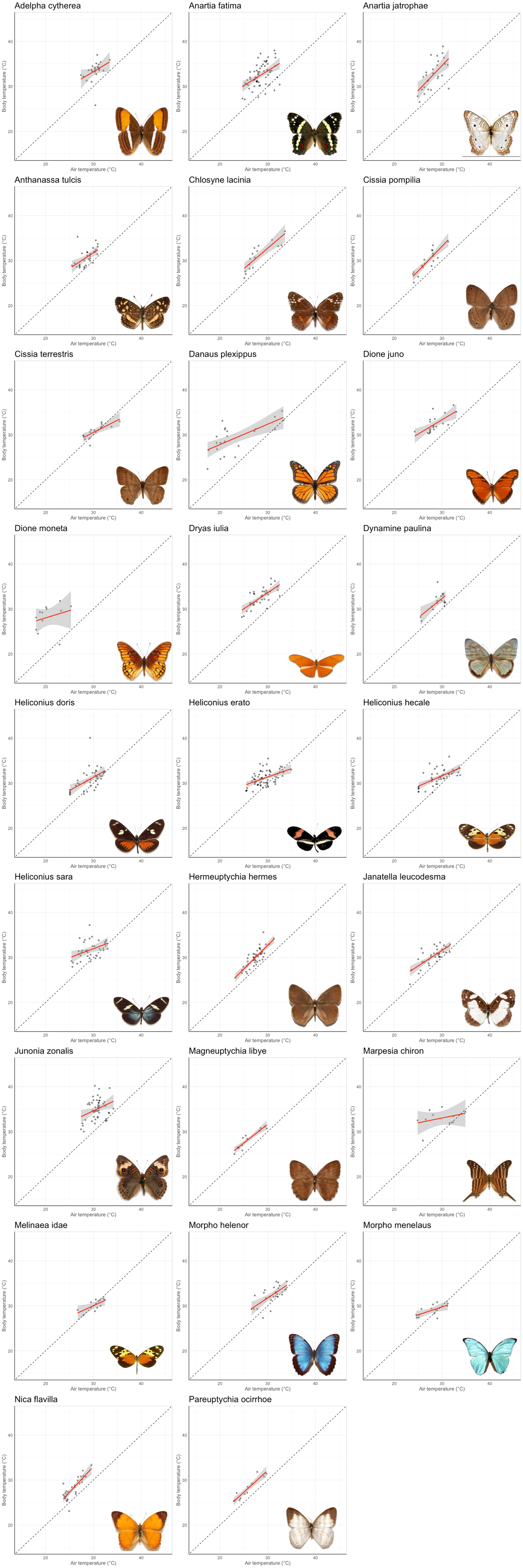


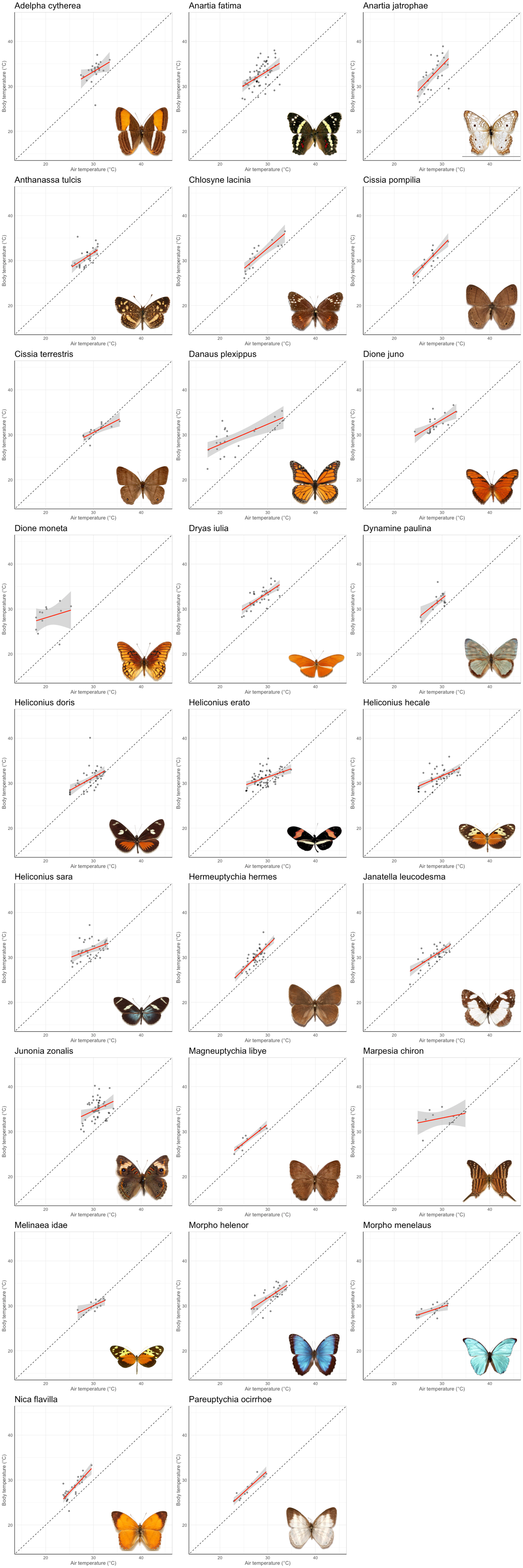


Figure S3: The relationship between body temperature (°C) and air temperature (°C) for 26 species of Nymphalidae. Points show individual butterflies. Red lines show the linear relationship between air and body temperature. Shaded areas show 95% confidence intervals. Black lines show a 1:1 relationship to aid visual comparison between species. For inset photo credits, see Table S2.


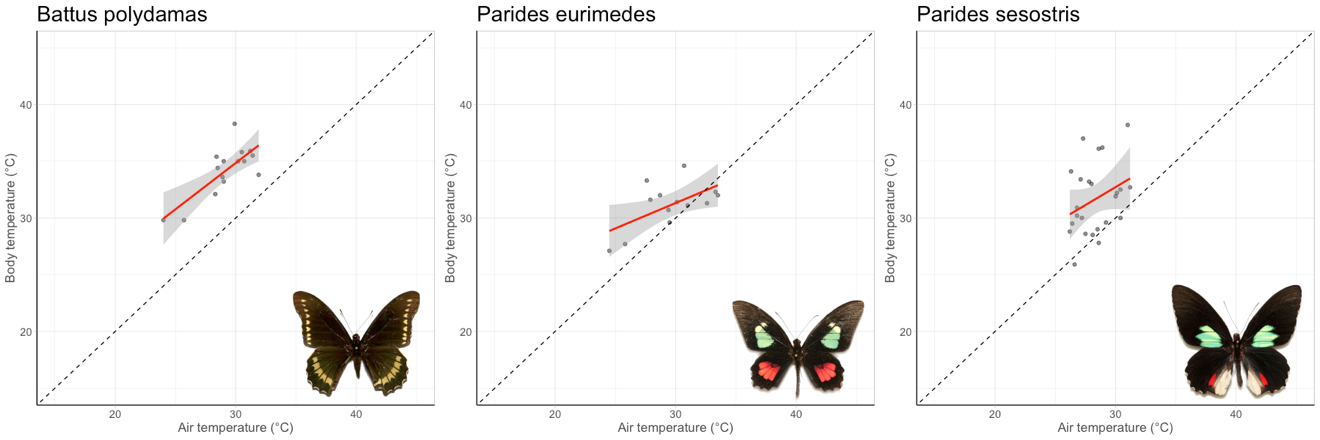


Figure S4: The relationship between body temperature (°C) and air temperature (°C) for three species of Papilionidae. Points show individual butterflies. Red lines show the linear relationship between air and body temperature. Shaded areas show 95% confidence intervals. Black lines show a 1:1 relationship to aid visual comparison between species. For inset photo credits, see Table S2.


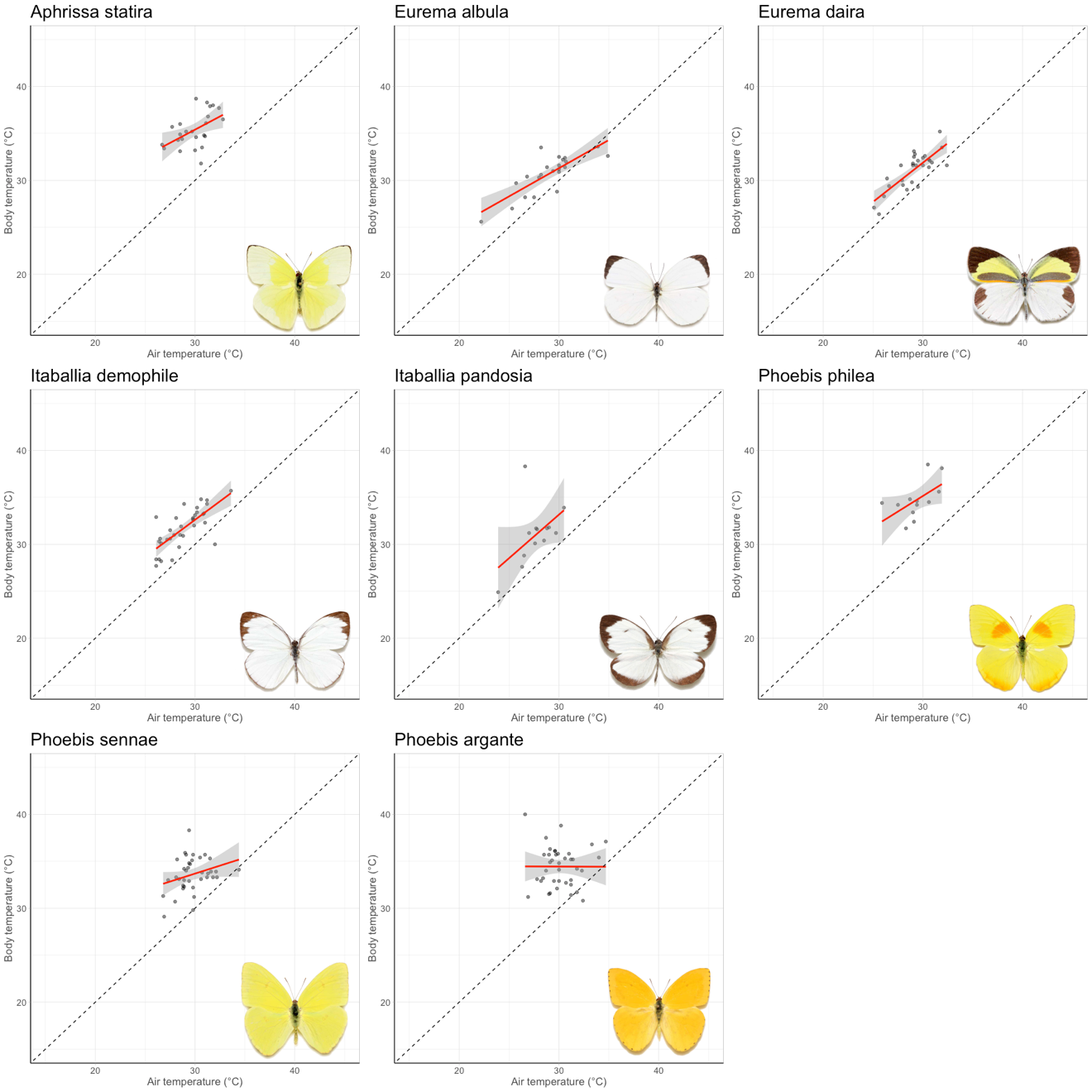


Figure S5: The relationship between body temperature (°C) and air temperature (°C) for eight species of Pieridae. Points show individual butterflies. Red lines show the linear relationship between air and body temperature. Shaded areas show 95% confidence intervals. Black lines show a 1:1 relationship to aid visual comparison between species. For inset photo credits, see Table S2.


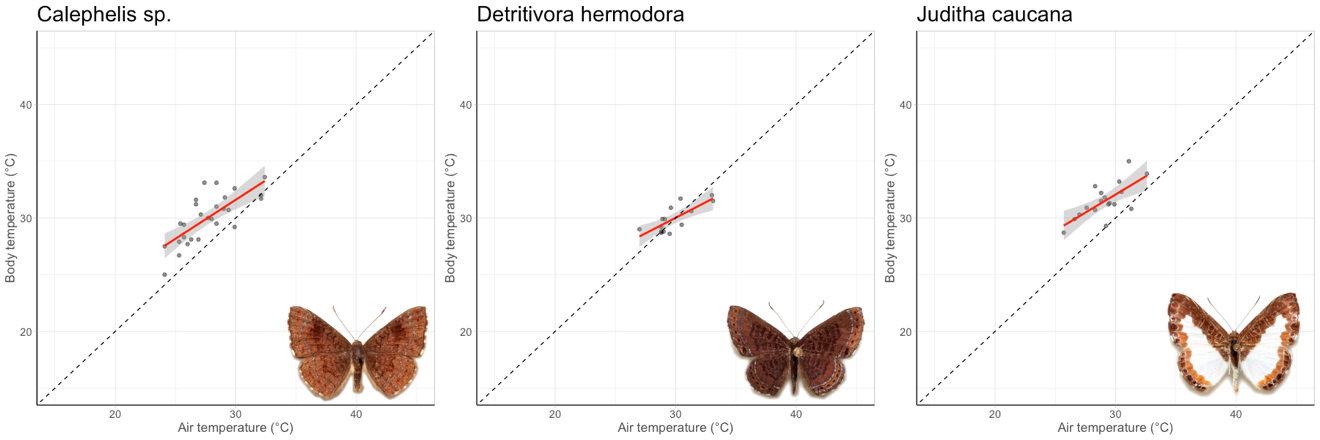


Figure S6: The relationship between body temperature (°C) and air temperature (°C) for three species of Riodinidae. Points show individual butterflies. Red lines show the linear relationship between air and body temperature. Shaded areas show 95% confidence intervals. Black lines show a 1:1 relationship to aid visual comparison between species. For inset photo credits, see Table S2.

Table S2: List of species with photo credits for insets used in Figs S1-7 and Table S5.

| **Species** | **Photo credit** |
| --- | --- |
| *Aides dysoni* | Bernard Hermier, © Images copyright Trustees Natural History Museum, used with permission |
| *Burnsius orcus* | Kim Davis, Mike Strangeland, Andrew Warren 2009 |
| *Cecropterus dorantes* | Kim Davis, Mike Strangeland, Andrew Warren 2009 |
| *Hylephila phyleus* | Jim P. Brock 2009 |
| *Pompeius pompeius* | Andrew Warren 2007 |
| *Spicauda procne* | Kim Davis, Mike Strangeland, Andrew Warren 2009 |
| *Spicauda simplicius* | Nick V. Grishin 2013 |
| *Staphylus ascalaphus* | Kim Davis, Mike Strangeland, Andrew Warren 2009 |
| *Staphylus azteca* | D.H. Janzen, W. Hallwachs 2010 |
| *Staphylus vulgata* | D.H. Janzen, W. Hallwachs 2010 |
| *Vehilius stictomenes* | Bernard Hermier, © Images copyright Trustees Natural History Museum, used with permission |
| *Arawacus togarna* | Kim Davis, Mike Strangeland, Andrew Warren 2008 |
| *Calycopis isobeon* | Jim P. Brock 2009 |
| *Cupido comyntas* | Kim Davis, Mike Strangeland, Andrew Warren 2010 |
| *Hemiargus hanno* | Kim Davis, Mike Strangeland, Andrew Warren 2009 |
| *Adelpha cytherea* | Kim Davis, Mike Strangeland, Andrew Warren 2009 |
| *Anartia fatima* | D.H. Janzen, W. Hallwachs 2011 |
| *Anartia jatrophae* | Kim Davis, Mike Strangeland, Andrew Warren 2009 |
| *Anthanassa tulcis* | Jim P. Brock 2009 |
| *Chlosyne lacinia* | Kim Davis, Mike Strangeland 2006 |
| *Cissia pompilia* | Kim Davis, Mike Strangeland 2006 |
| *Cissia terrestris* | Nick V. Grishin 2010 |
| *Danaus plexippus* | Andrew Warren 2011 |
| *Dione juno* | Kim Davis, Mike Strangeland 2005 |
| *Dione moneta* | Andrew Warren 2010 |
| *Dryas iulia* | Kim Davis, Mike Strangeland, Andrew Warren 2009 |
| *Dynamine paulina* | Kim Davis, Mike Strangeland, Andrew Warren 2009 |
| *Heliconius doris* | Kim Davis, Mike Strangeland, Andrew Warren 2010 |
| *Heliconius erato* | Gerardo Lamas, © Images copyright Trustees Natural History Museum, used with permission |
| *Heliconius hecale* | Kim Davis, Mike Strangeland, Andrew Warren 2009 |
| *Heliconius sara* | Kim Davis, Mike Strangeland, Andrew Warren 2009 |
| *Hermeuptychia hermes* | Kim Davis, Mike Strangeland 2006 |
| *Janatella leucodesma* | Kim Davis, Mike Strangeland, Andrew Warren 2009 |
| *Junonia zonalis* | John Calhoun 2010 |
| *Magneuptychia libye* | Jim P. Brock 2011 |
| *Marpesia chiron* | Andrew Warren 2011 |
| *Melinaea idae* | Keith Willmott 2010 |
| *Morpho helenor* | Kim Davis, Mike Strangeland, Andrew Warren 2009 |
| *Morpho menelaus* | Kim Davis, Mike Strangeland, Andrew Warren 2009 |
| *Nica flavilla* | Kim Davis, Mike Strangeland, Andrew Warren 2009 |
| *Pareuptychia ocirrhoe* | Kim Davis, Mike Strangeland 2010 |
| *Battus polydamas* | Kim Davis, Mike Strangeland 2005 |
| *Parides eurimedes* | Jim P. Brock 2009 |
| *Parides sesostris* | Kim Davis, Mike Strangeland, Andrew Warren 2008 |
| *Aphrissa statira* | Andrew D. Warren 2011 |
| *Eurema albula* | Andrew D. Warren 2011 |
| *Eurema daira* | Kim Davis, Mike Strangeland, Andrew Warren 2009 |
| *Itaballia demophile* | Kim Davis, Mike Strangeland, Andrew Warren 2010 |
| *Itaballia pandosia* | Kim Davis, Mike Strangeland, Andrew Warren 2009 |
| *Phoebis argante* | Andrew D. Warren 2011 |
| *Phoebis philea* | Kim Davis, Mike Strangeland, Andrew Warren 2009 |
| *Phoebis sennae* | Andrew D. Warren 2011 |
| *Calephelis sp* | Kim Davis, Mike Strangeland, Andrew Warren 2008 |
| *Detritivora hermodora* | Kim Davis, Mike Strangeland, Andrew Warren 2008 |
| *Juditha caucana* | Kim Davis, Mike Strangeland, Andrew Warren 2010 |

Table S3: Mixed effect linear model results, with all fixed effects and interaction effects (denoted by a colon between fixed effects) listed. Significant p-values are in bold. As all interaction terms were significant in the full model, no model selection was necessary.

| **Term** | **Chi sq** | **D.f.** | **P-value** |
| --- | --- | --- | --- |
| Air temperature | 726.15 | 1 | **< 0.001** |
| Family | 19.4 | 5 | **0.002** |
| Wing length | 9.17 | 1 | **0.002** |
| Wing colour | 0.18 | 1 | 0.671 |
| Air temperature:Family | 12.96 | 5 | **0.024** |
| Air temperature:Wing length | 16.33 | 1 | **< 0.001** |
| Air temperature:Wing colour | 4.70 | 1 | **0.030** |

Table S4: The intercepts and slopes (± 1 standard error) for each term in the mixed effect linear model. Slope estimates for each family indicate the interaction with air temperature.

| **Term** | **Intercept** | **Slope** |
| --- | --- | --- |
| Hesperiidae | -4.90 ± 4.64 | 1.31 ± 0.16 |
| Lycaenidae | -9.04 ± 4.08 | 1.42 ± 0.14 |
| Nymphalidae | -3.25 ± 2.29 | 1.18 ± 0.08 |
| Papilionidae | -6.36 ± 4.50 | 1.31 ± 0.15 |
| Pieridae | 4.38 ± 3.87 | 0.95 ± 0.13 |
| Riodinidae | 0.79 ± 4.04 | 1.03 ± 0.14 |
| Wing length | -4.90 ± 4.64 | 0.31 ± 0.07 |
| Wing colour | -4.90 ± 4.64 | 1.68 ± 0.85 |

Table S5: List of 24 species included in thermal tolerance analyses, ordered alphabetically by family, with the temperature at which 50% of individuals were knocked down (LD50), and the difference between the temperatures at which 90% were still standing and the temperature at which 10% were still standing (knock down range). The number of individuals per species is also shown (sample size). Two species followed by * were excluded from analysis of thermal buffering ability and thermal tolerance due to insufficient thermal buffering data. For inset photo credits, see Table S2.

| **Photo** | **Family** | **Species** | **LD50** | **Knock down range** | **Sample size** |
| --- | --- | --- | --- | --- | --- |
| 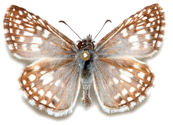 | Hesperiidae | *Burnsius orcus* | 51.0 | 12.3 | 21 |
| 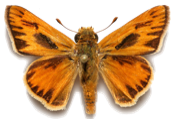 | Hesperiidae | *Hylephila phyleus* | 51.7 | 9.6 | 20 |
| 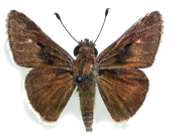 | Hesperiidae | *Pompeius pompeius* | 52.3 | 10.8 | 21 |
| 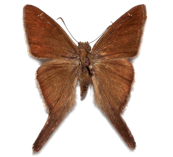 | Hesperiidae | *Spicauda procne* | 52.4 | 15.1 | 20 |
| 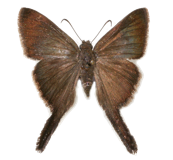 | Hesperiidae | *Spicauda simplicius* | 52.1 | 8.1 | 20 |
| 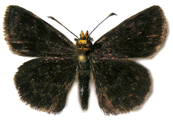 | Hesperiidae | *Staphylus vulgata* | 46.5 | 11.3 | 20 |
| 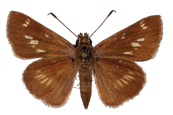 | Hesperiidae | *Vehilius stictomenes* | 51.7 | 10 | 21 |
| 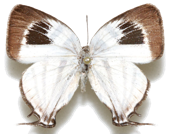 | Lycaenidae | *Arawacus togarna* | 49.7 | 8.8 | 18 |
| 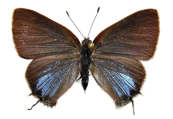 | Lyceanidae | *Calycopis isobeon** | 46.5 | 10.9 | 20 |
| 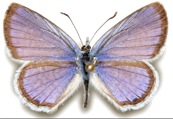 | Lycaenidae | *Hemiargus hanno* | 49.5 | 11.9 | 23 |
| 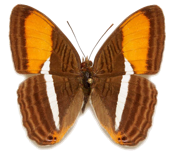 | Nymphalidae | *Adelpha cytherea* | 47.1 | 6.2 | 20 |
| 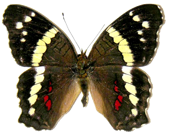 | Nymphalidae | *Anartia fatima* | 48.7 | 9.2 | 22 |
| 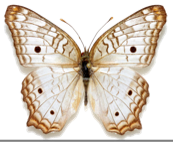 | Nymphalidae | *Anartia jatrophae* | 52.7 | 10.2 | 21 |
| 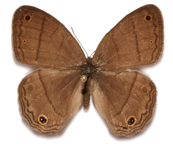 | Nymphalidae | *Cissia pompilia* | 46.3 | 4.9 | 20 |
| 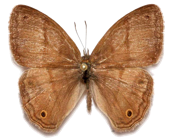 | Nymphalidae | *Cissia pseudoconfusa** | 51.1 | 7.9 | 21 |
| 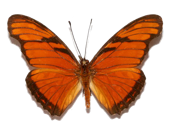 | Nymphalidae | *Dione juno* | 45.2 | 2.5 | 20 |
| 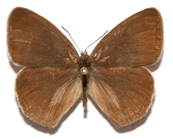 | Nymphalidae | *Hermeuptychia hermes* | 50.2 | 10.2 | 20 |
| 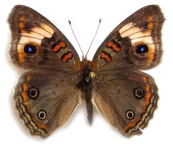 | Nymphalidae | *Junonia zonalis* | 56.8 | 12.5 | 20 |
| 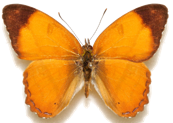 | Nymphalidae | *Nica flavilla* | 47.5 | 5.3 | 22 |
| 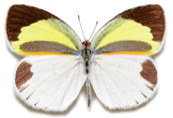 | Pieridae | *Eurema daira* | 52.2 | 8.4 | 22 |
| 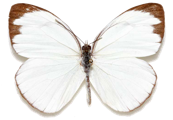 | Pieridae | *Itaballia demophile* | 45.1 | 5.9 | 24 |
| 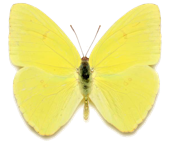 | Pieridae | *Phoebis sennae* | 49.3 | 5.1 | 21 |
| 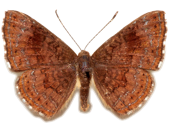 | Riodinidae | *Calephelis* sp. | 49.9 | 10.9 | 21 |
| 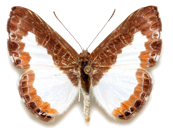 | Riodinidae | *Juditha caucana* | 45.8 | 4.6 | 21 |


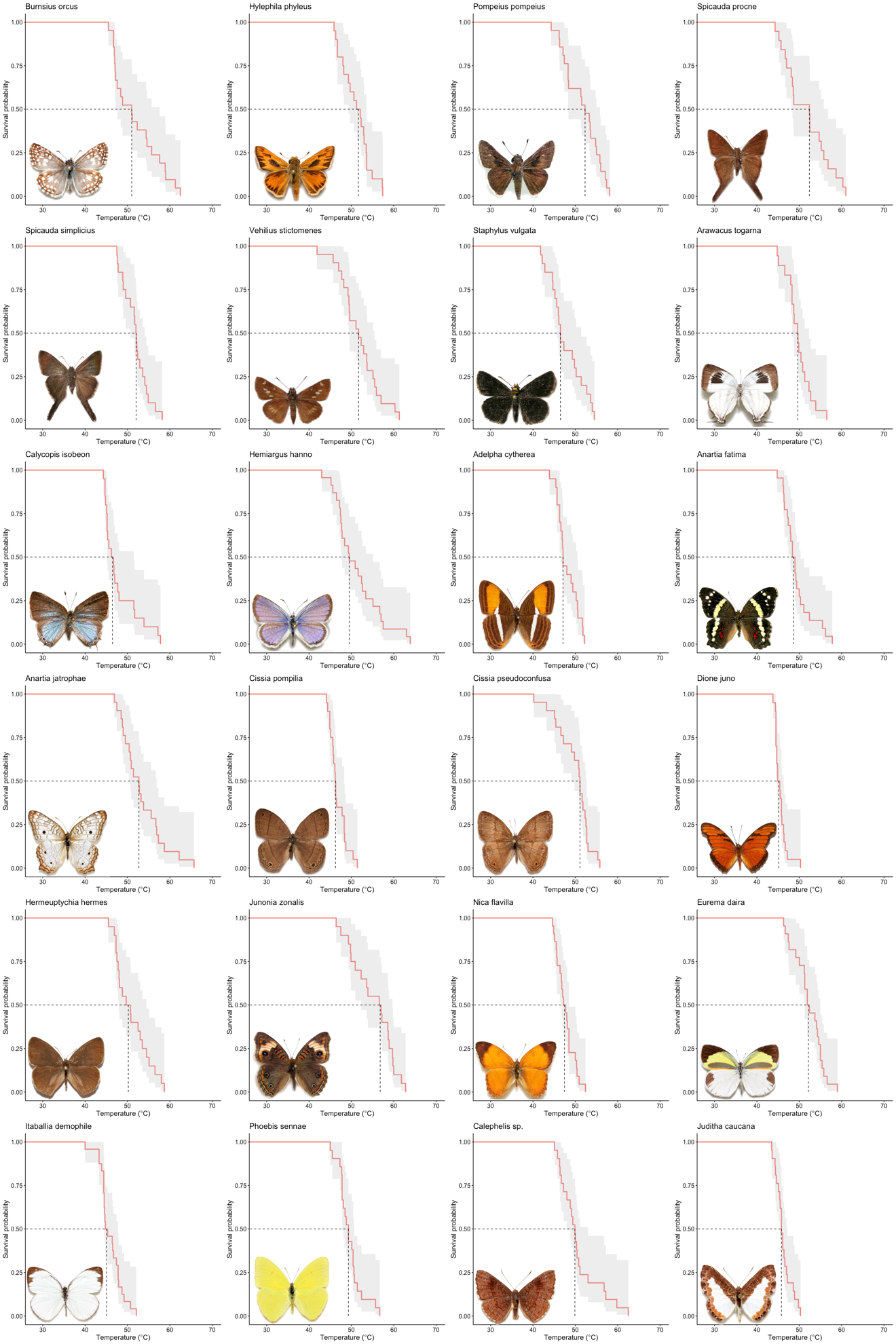


Figure S7: Average thermal survival curves for 24 species. Temperature was increased from 28°C to 70°C at 0.5°C per minute until the butterfly was knocked down (see Methods). Red solid lines show mean survival, ribbons represent 95% confidence intervals. Dashed lines show the temperature at which 50% of individuals were knocked down (LD50) per species. Species are ordered alphabetically by family. For inset photo credits, see Table S2.


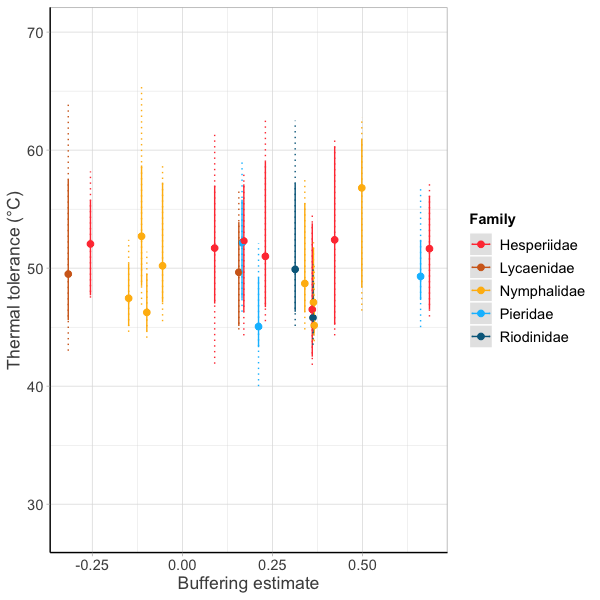


Figure S8: The relationship between species-specific buffering estimate and thermal tolerance of 22 species for which both values were calculated (two species that had their thermal tolerance calculated did not have sufficient data to calculate thermal buffering ability and were excluded from this analysis). Points represent the average temperature at which 50% of individuals were knocked down (LD50). Solid lines represent the range within species between which 10% and 90% of individuals were knocked down (the difference between which is the knock down range). Dotted lines represent the total temperature range within species from the first to last knocked down individual. Points are coloured by family, and have been jittered to make overlaying points visible.
